# Accounting Gut Microbiota as the Mediator of Beneficial Effects of Dietary (Poly)phenols on Skeletal Muscle in Aging

**DOI:** 10.3390/nu15102367

**Published:** 2023-05-18

**Authors:** Andrea Ticinesi, Antonio Nouvenne, Nicoletta Cerundolo, Alberto Parise, Tiziana Meschi

**Affiliations:** 1 Department of Medicine and Surgery, University of Parma, Via Antonio Gramsci 14, 43126 Parma, Italy; tiziana.meschi@unipr.it; 2 Microbiome Research Hub, University of Parma, Parco Area delle Scienze 11/1, 43124 Parma, Italy; anouvenne@ao.pr.it; 3 Geriatric-Rehabilitation Department, Azienda Ospedaliero-Universitaria di Parma, Via Antonio Gramsci 14, 43126 Parma, Italy; ncerundolo@ao.pr.it (N.C.); aparise@ao.pr.it (A.P.)

**Keywords:** sarcopenia, physical frailty, curcumin, isoflavones, anthocyanins, flavonoids

## Abstract

Sarcopenia, the age-related loss of muscle mass and function increasing the risk of disability and adverse outcomes in older people, is substantially influenced by dietary habits. Several studies from animal models of aging and muscle wasting indicate that the intake of specific polyphenol compounds can be associated with myoprotective effects, and improvements in muscle strength and performance. Such findings have also been confirmed in a smaller number of human studies. However, in the gut lumen, dietary polyphenols undergo extensive biotransformation by gut microbiota into a wide range of bioactive compounds, which substantially contribute to bioactivity on skeletal muscle. Thus, the beneficial effects of polyphenols may consistently vary across individuals, depending on the composition and metabolic functionality of gut bacterial communities. The understanding of such variability has recently been improved. For example, resveratrol and urolithin interaction with the microbiota can produce different biological effects according to the microbiota metabotype. In older individuals, the gut microbiota is frequently characterized by dysbiosis, overrepresentation of opportunistic pathogens, and increased inter-individual variability, which may contribute to increasing the variability of biological actions of phenolic compounds at the skeletal muscle level. These interactions should be taken into great consideration for designing effective nutritional strategies to counteract sarcopenia.

## 1. Introduction

Sarcopenia is a geriatric syndrome with high prevalence in the older population, characterized by a loss of muscle mass and function secondary to a chronic illness or in absence of any identifiable underlying cause [1,2]. This condition is frequently overlapped with frailty and multimorbidity [3], and is associated with a relevant risk of adverse outcomes, including disability, institutionalization, hospitalization and mortality [4].

The pathogenesis of sarcopenia is multifactorial and involves multiple mechanisms, including malnutrition with reduced amino acid availability, insulin resistance, anabolic resistance and chronic inflammation [5,6]. All these pathways lead to myocellular mitochondrial dysfunction and reduced muscle protein synthesis with enhanced catabolism [7,8].

The gut microbiota, i.e., the ensemble of microorganisms symbiotically living with the host in the gut lumen, is potentially able to influence all these mechanisms leading to muscle wasting and loss of function [9,10,11]. Therefore, several researchers have hypothesized the existence of a “gut-muscle axis” influences the onset of sarcopenia in older individuals [9,10,11], especially following the age-related changes in gut microbiota composition and function [12]. In the extreme ages of life, in fact, an imbalance between the representation of symbiotic microorganisms and opportunistic pathogens generally occurs, with potentially negative consequences for the host [13]. A recent systematic review of the studies comparing the gut microbiota composition of sarcopenic vs. non-sarcopenic older subjects has confirmed that the presence of sarcopenia is associated with a distinct microbiota composition characterized by an overrepresentation of pathogenic bacteria [14].

One of the main therapeutical strategies proposed to counteract sarcopenia in older people is promotion of a healthy diet with balanced intake of proteins with a high biological value [15,16]. The paradigm of such a healthy diet is represented by the Mediterranean-style diet [17], which, in aging, is inversely associated with loss of muscle function, and probably, also with muscle wasting, although this association is still debated [18,19,20,21].

Mediterranean diet is rich of foods of vegetal origin with a high polyphenol content. Polyphenols are non-nutrient bioactive compounds that exert pleiotropic physiological functions after absorption and biotransformation by phase-I and phase-II enzymes [22,23]. Namely, several in vitro and preclinical studies have shown that polyphenolic metabolites have also beneficial effects for skeletal muscle cells, and thus a protective action against muscle wasting [24]. Diets rich in fruit, vegetables and other foods of vegetal origin with high polyphenol content are thus increasingly regarded as a promising non-pharmacologic therapeutical strategy against sarcopenia [25,26].

However, recent studies have contributed to elucidate that gut microbial metabolism is also deeply involved in the biotransformation of dietary polyphenols into bioactive compounds [27]. Therefore, the myoprotective action of dietary polyphenols could, at least partly, rely on gut microbiota composition and functionality [27]. In older age, the intestinal microbiota is characterized by a tendency towards dysbiosis and an increased inter-individual variability [28], whose impact on the metabolism of dietary bioactives is still poorly investigated.

The aim of this narrative review is to disentanzgle the complex relationship linking diet, microbiota and skeletal muscle in the older age, discuss the relevance of gut microbiota as mediator of the myoprotective effects of the main polyphenolic compounds, and identify possible lines of future research with relevance for geriatric medicine.

A literature search was conducted on PubMed as of 31 March 2023, following a strategy that comprises multiple queries which identified articles containing the name of specific phenolic subclasses (for example, “flavonoids”, “flavones”, or “anthocyanins”) or the name of single compounds (for example, “urolithin”, “genistein”, or “resveratrol”) as keywords, in association with “microbiome” or “gut microbiota” and at least one of the following: “sarcopenia”, “physical frailty”, “muscle wasting”, “muscle mass”, “muscle function”, “dynapenia”, and “fatigue”. Articles were then screened for their relevance with the primary aim of the present narrative review, and only those reporting research conducted in older human subjects or research contributing to explain the relevant mechanisms of the complex interaction between (poly)phenols, microbiota and sarcopenia in older patients, were included for discussion.

## 2. Overview of Polyphenolic Compounds with Potential Myoprotective Action

Experimental studies, conducted in vitro or in animal models, have shown that several phenolic compounds contained in foods of vegetal origin can exert protective effects for skeletal muscle cells through multiple mechanisms. An overview of these effects, recently reviewed in an extended way by Nikawa and colleagues [24], is provided in Table 1, in accordance with the current taxonomical classification of (poly)phenolic compounds [29].

All the phenolic compounds listed in Table 1 have pleiotropic actions, and may thus exert significant physiological actions not limited just to skeletal muscle or myotubules, but also to other organs and systems, including the gastrointestinal and central nervous system [29]. Phenolic compounds, in fact, share health-promoting claims that do not depend only on their antioxidant properties, but also on their capacity of regulating mitochondrial biogenesis and function, balancing protein synthesis and degradation, and modulating cellular pathways involved in cell differentiation or apoptosis [30,31,32,33,34,35,36,37,38,39,40,41,42,43,44,45,46,47]. These effects have been studied more in depth for a few phenolic compounds, including favanols (epicatechins and derivatives) [35,36], soy isoflavones (genistein and daidzein) [40], quercetin [42], resveratrol [44] and curcumin [47], while for other compounds, the comprehension of physiological functions is still at the beginning.

The precise biochemical mechanisms by which (poly)phenolic compounds may exert protective actions on skeletal muscle cells are not fully understood and are the object of ongoing research. Many phenolic compounds exert their protective functions at the intracellular level, through direct or indirect interaction with transcriptional (such as PGC-1α, Nrf1, TFAM) or regulatory factors (such as myogenin, Myf5, MyoD) [24]. The capacity of activating SIRT-1 seems central in these complex pathways [24]. Furthermore, polyphenolic compounds downregulate factors involved in promoting inflammation such as NF-κB, TNF-α and cyclooxygenase-2 (COX-2), as well as factors involved in protein degradation such as ubiquitin ligases, atrogin-1, and myostatin [24]. Downregulation of the TGF-β/myostatin-Akt-mTORC pathway and upregulation of enzymes with pivotal antioxidant functions, such as superoxide dismutase and catalase, which are also important mechanisms [24]. There are also phenolic compounds interacting with endocrine receptors, such as isoflavones that mimic estrogenic functions by activating the estrogen receptors Erα and ERβ [24].

However, experimental studies hardly ever consider the complex biotransformation pathways that each polyphenolic compound undergo within the human body, partly dependent on intestinal and liver metabolism, and partly dependent on gut microbiota metabolism [27]. Most biological functions of the compounds listed in Table 1, in fact, depend on their metabolites produced by liver and gut bacterial metabolism [27]. Interestingly, patients suffering from cirrhosis exhibit an increased risk of sarcopenia with a high probability of an extreme loss of muscle mass and function [48,49]. Severe impairment of the capacity of transforming dietary (poly)phenols in bioactive compounds with myoprotective actions, due to reduced liver function and associated gut microbiota dysbiosis, may therefore be involved in the pathophysiology not only of cirrhosis-associated sarcopenia, but also of age-related sarcopenia. Gut microbiota dysbiosis and reduced liver function are in fact commonplace in frail older patients at risk for sarcopenia, and the reduced capacity of transforming dietary (poly)phenols into bioactive compounds has been postulated as one of the central mechanisms of the gut-muscle axis leading to muscle wasting and sarcopenia in the older age [50,51].

## 3. Interaction between Phenolic Compounds and Microbiota: Possible Relevance for Sarcopenia

### 3.1. Ellagitannins and Derivatives

Ellagitannins are hydrolysable tannins that release ellagic acid or its derivative, gallic acid, which is frequently found in nuts, pomegranates and berries. The hydrolyzation process can occur spontaneously in acid pH during digestion, but may also be triggered by gut microbial species, including *Akkermansia muciniphila* [27,52,53]. Ellagic acid has a very low bioavailability in the intestinal lumen, due to its idrophobic nature, and can be metabolized into urolithin-M5: the precursor of the compounds belonging to the urolitin family by specific gut microbiota functionalities [53]. The first bacterial species identified as able to carry on this metabolic step were *Gordonibacter pamelae* and *Gordonibacter urolilithinfaciens* [54]. More recently, other bacterial taxa, more commonly found in the human gut microbiota, have been identified as able to synthetize urolithin-M5, including Eggerthellaceae members, *Lactobacillus*, *Leuconostoc* and *Pedicococcus* [55]. The further steps of the metabolic pathway, leading to the synthesis of the metabolically active compounds urolithin A and urolithin B, are still poorly known [27].

However, after a trial of administration of foods with high ellagitannin content, at least three different metabotypes can be identified in human beings, so that the final metabolites of the pathway can be produced only in the presence of specific gut microbiota functionalities [56]. Ellagic acid metabolizers can produce urolithin-A or urolithin-B (Uro-A or Uro-B metabotypes, respectively) [57], two compounds with myoprotective functions [58,59,60]. Urolithin A, in particular, has been recently proven effective in improving muscle strength and exercise endurance in human beings [59,60]. Subjects with Uro-0 metabotype, instead, do not produce bioactive urolithins after ingestion of foods rich in ellagitannins and ellagic acid [57]. Therefore, the beneficial properties of ellagitannins for host health in general, and muscle health in particular, substantially depend on gut microbiota composition and functionality [61].

Therefore, to benefit from dietary intake of foods rich in ellagitannins at the skeletal muscle level, older subjects should have a good representation in their gut microbiome of *Akkermansia muciniphila* and *Lactobacillus* spp., among others. Interestingly, *Akkermansia muciniphila* has been identified as one of the main bacterial taxa whose representation in the gut microbiota was associated with longevity in centenarians and subjects with a successful aging pattern [12,62], and was identified as a key species promoting healthy aging in animal models [63]. The abundance of *Akkermansia* was also associated with muscle mass in a study evaluating the microbiota composition of subjects with active or sedentary lifestyle [64]. Conversely, its underrepresentation was identified as a marker of sarcopenia in patients with cirrhosis [65]. However, in patients with chronic kidney disease (CKD)-associated sarcopenia, *Akkermansia* abundance was increased in comparison with patients with normal muscle mass [66]. Similarly, in a study investigating the associations of gut microbiota with frailty, *Akkermansia* had an increased representation in subjects with poorer physical performance [67].

The administration of probiotics with bacteria of the *Lactobacillus* genus is associated with increased muscle mass and strength, according to a recent systematic review and meta-analysis [68]. However, in a study comparing the gut microbiota composition of 27 patients with established or possible sarcopenia and 60 non-sarcopenic controls, the abundance of *Lactobacillus* spp. was increased, and not decreased [69].

These findings suggest that the microbiota urolithin metabotype cannot be established a priori, even in older subjects suffering from sarcopenia and with gut microbiota alterations typical of dysbiosis. The beneficial effects of ellagitannin ingestion on skeletal muscle health may thus show consistent inter-individual variability in older subjects. The administration of nutritional supplements containing ellagitannins may therefore provide clinically relevant benefits for muscle health only in subjects with favorable metabotypes, and do not provide any physiological effects at all in other individuals. Furthermore, older subjects with marked gut microbiota dysbiosis and reduced representation of *Lactobacillus* or *Akkermansia* may show limited benefits from ellagitannin administration as well. The co-administration of probiotics with blends containing Lactobacilli and ellagitannins could contribute to counteract the effects of dysbiosis on urolithin formation, but no studies have assessed this hypothesis to date.

### 3.2. Hydroxycinnamic Acid Derivatives

Chlorogenic acid is the phenolic compound of this category most frequently found in foods, including several fruits, coffee, potatoes and artichokes [32,33,34]. Ferulic acid and caffeic acid can be either less frequently found in foods of vegetal origin, or produced by the gut microbiota biotransformation of chlorogenic acid through two distinct metabolic pathways [70,71]. The bacterial species involved and the associated gut microbiota metabotypes are far less known than what happens for ellagitannins. The existing studies, in fact, were focused on metabolic transformations and not on microbiota [70,71]. However, there is substantial agreement that *Bifidobacterium* spp. strains are strongly involved in these pathways [70,71].

Chlorogenic acid and its derivatives can modulate skeletal muscle physiology, exerting myoprotective actions in multiple ways: regulating muscle fiber type formation [72], sustaining capillarization of muscle tissue [73], promoting myocellular glucose uptake [74,75], reducing oxidative stress [74,76], modulating protein synthesis, and preventing mitochondrial dysfunction [77]. Hippuric acid, another metabolite resulting from chlorogenic acid biotransformation by gut microbiota and host metabolism, may also exert myoprotective functions [78,79]. Its role as possible biomarker of frailty and sarcopenia in older subjects has been recently reviewed by our research group [26].

Although no specific studies have assessed this issue, it seems plausible that the positive effects against muscle wasting of hydroxycinnamic acid derivatives are emphasized only in presence of an adequate representation of *Bifidobacterium* spp. in the gut microbiota. The maintenance of a core population of Bifidobacteria has been associated with longevity and successful aging, being one of the hallmarks of gut microbiota in centenarians [62]. Interestingly, a recent study conducted in 50 older Chinese patients with sarcopenia and 50 controls has identified the depletion of *Bifidobacterium longum* as one of the main microbial biomarkers associated with sarcopenia [80]. The administration of probiotics containing bifidobacterial strains has also been recently identified as effective in improving muscle mass and strength in human beings of different ages, either with or without sarcopenia, according to a recent systematic review and meta-analysis [68]. *Bifidobacterium longum* probiotic strains, in particular, are characterized by an extensive capacity of establishing cooperative interactions with other members of gut bacterial community, and for promoting integrity of the gut mucosa [81], a fundamental mechanism for limiting inflammatory pathways leading to sarcopenia. However, in another study conducted in 35 older community-dwellers from Italy, either with physical frailty and sarcopenia or with normal muscle mass and function, the abundance of *Bifidobacterium* spp. was associated with reduced muscle performance [82]. Therefore, the role of Bifidobacteria in the gut-muscle axis is still far from understood, and the response to dietary intake of hydroxycinnamic acid derivatives may substantially vary across older individuals even with similar muscle mass and function.

The current state of knowledge, however, supports the hypothesis that the myoprotective actions of hydroxycinnamic acid derivatives are less pronounced in older subjects with a reduced representation of Bifidobacteria in their microbiota. The co-administration of nutritional supplements containing chlorogenic acid or its derivatives with bifidobacterial species as probiotics could contribute to improve the anti-sarcopenic effects of this phenolic subclass.

### 3.3. Proanthocyanidins and Flavan-3-ols (Flavanols)

Flavan-3-ols (flavanols) are phenolic compounds derived from flavans, and frequently found in berries, grapes, cocoa, plums and tea [24,27]. The most known compounds include epicatechin, epigallocatechin and epigallocatechin gallate. Proanthocyanidins are oligomers of epicatechin, epigallocatechin and their gallic acid esters, frequently found in several fruits and particularly in berries and grapes [24,27].

The flavanols contained in foods in the monomeric form and oligomeric proanthocyanidins are generally subject to host metabolism in enterocytes and epatocytes, undergoing glucuronidation or sulfonation independently of gut microbiota [83,84]. The formed metabolites of epicatechin, epigallocatechin or epigallocatechin gallate may exert protective effects on skeletal muscle cells. Epicatechin, in particular, can represent a powerful modulator of AMPK and Akt/mTOR pathways leading to increased protein synthesis [85,86]. Epicatechin is also able to inhibit the TLR/NF-κB pathway of inflammatory response, counteract reactive oxygen species (ROS) formation, and promote mitochondrial biogenesis in experimental models [85,86,87]. Finally, there is also evidence of an activation of muscle stem cells mediated by epicatechin, promoting muscle regeneration [88]. Interestingly, a combined intervention consisting of epicatechin supplementation, plus regular resistance training, resulted in improvements in muscle strength in a group of sarcopenic older individuals [89]. The beneficial effects of epigallocatechin and epigallocatechin gallate for skeletal muscle cells are less established, but experimental and in vitro research indicate that they may be protective against the onset of muscle wasting related to disuse [90,91,92,93].

Unlike monomeric flavanols and oligomeric proanthocyanidins, polymeric proanthocyanidins, accounting for >90% of the dietary compounds belonging to this phenolic subclass, undergo relevant metabolism at the gut microbiota level [83,84]. Specific gut microbiota functionalities, in fact, may promote degradation of polymeric proanthocyanidins into flavanols or oligomeric compounds absorbable by the intestinal mucosa [94,95]. Alternatively, they can transform proanthocyanidins into phenyl-valerolactones and derivatives [94,95]. These compounds have antioxidant and antihypertensive properties, but their specific action on skeletal muscle cells has not been comprehensively investigated to date [27]. One study conducted in mice, however, suggests that phenyl-γ-valerolactones may promote glucose uptake through GLUT4 transporter and favor protein synthesis in skeletal muscle cells [96].

The bacterial taxa involved in proanthocyanidins metabolism in the gut microenvironment are also uncertain. *Clostridium coccoides*, *Bifidobacterium infantis*, *Eggerthella lenta* and *Adlercreutzia equolifaciens* are among the most probable candidates [97,98,99]. Among these taxa, *Eggerthella lenta* has been recognized as one of the main microbial biomarkers of frailty. Its abundance was in fact positively associated with the Frailty Index in a large group of older female twins from the TwinsUK cohort [100] and in a smaller study conducted in community-dwellers from the US [101]. *Eggerthella lenta* was also positively associated with sarcopenia and altered body composition in patients with cirrhosis [35,102]. Finally, *Adlercreutzia* spp. Abundance in fecal samples of 373 older community-dwelling men from the US was inversely associated with the level of habitual physical activity, suggesting that this bacterial taxon may represent a marker of unhealthy lifestyle in aging [103]. Overall, these results support the hypothesis that older subjects with physical frailty, sarcopenia, and a predominantly sedentary lifestyle, may have a higher efficiency in metabolizing proanthocyanidins into bioactive compounds with myoprotective action than subjects without frailty and sarcopenia. Thus, proanthocyanidins may represent very promising candidates as nutritional supplements tailored at preserving muscle health in the older aged. Unfortunately, no study has specifically addressed this issue to date. However, in a randomized controlled trial conducted in post-menopausal women, the administration of grape seed proanthocyanidins was associated with significant improvements in physical performance and muscle mass after eight weeks [104].

### 3.4. Flavanones

Flavanones are a class of phenolic compounds mainly contained in citrus fruits. Hesperitin, its glycosylated derivative hesperidin and naringenin, are the most studied compounds [37]. Hesperidin has shown several myoprotective actions in experimental models, modulating mitochondrial biogenesis and function, reducing ROS formation and local inflammation [105]. In a randomized controlled trial conducted in 40 amateur cyclists, dietary hesperidin supplementation was associated with increased muscle mass [106]. Naringenin can also increase glucose uptake in skeletal muscle, reduce myocellular diacylglycerol accumulation and promote myocellular differentiation by interaction with estrogen receptors α and β [107,108,109]. Furthermore, a derivative of naringenin, 8-prenylnaringenin, frequently found in hops and beer, has also shown myoprotective actions in experimental models [38].

Hesperidin has low intestinal bioavailability. To be absorbed by the gut mucosa, it must be converted in hesperitin and its derivative hesperitin 7-O-glucoside by specific gut microbiota functionalities that are harbored in Bifidobacteria, and particularly in *Bifidobacterium pseudocatenulatum*, which is a species producing the key enzyme for the biotransformation α-rhamnosidase [110,111]. Different metabotypes of hesperidin biotransformation can be identified in human beings, according to the presence of this enzyme by the gut microbiota and its representation [112].

The microbial pathways of naringenin biotransformation are even less understood, but the presence of Bifidobacteria with α-rhamnosidase functionalities seems to be pivotal for the synthesis of bioactive metabolites that can be absorbed by the gut mucosa [113,114]. The administration of a probiotic strain of *Bifidobacterium longum* producing α-rhamnosidase was in fact associated with an increased urinary excretion of naringenin metabolites after orange juice consumption [115]. Finally, intestinal biotransformation of 8-prenylnaringenin into absorbable and physiologically active compounds seems to depend on specific enzymatic functionalities harbored in *Eubacterium limosum* and *Eubacterium ramulus* [116,117].

In this context, an adequate representation of Bifidobacteria in gut microbial communities seems to be of paramount importance for mediating the myoprotective effects of flavanones. Therefore, as discussed in Section 3.2, older subjects with a healthy active aging pattern and good gut microbiota representation of Bifidobacteria are those who may benefit the most of the beneficial effects of flavanones on skeletal muscle mass. Conversely, older, frail subjects with a tendency towards gut microbiota dysbiosis and a reduced representation of *Bifidobacterium* may show reduced benefits from flavanone supplementation, although no specific study has assessed this issue to date. Probiotic interventions aimed at restoring an adequate population of Bifidobacteria in the gut microbiota may therefore be necessary before the effects of dietary flavanone supplementation against muscle wasting becomes evident in older individuals.

Regarding the myoprotective effects of 8-prenylnaringenin, a recent study has shown a reduced representation of *Eubacterium* spp. in older individuals with sarcopenia [69]. *Eubacterium limosum* abundance was also identified as a marker of the gut microbiota of centenarians [118]. Therefore, the beneficial effects of 8-prenylnaringenin derived from hops may be enhanced only in those individuals with a favorable aging pattern, and reduced in patients with physical frailty and sarcopenia.

### 3.5. Flavones

Flavones represent a subclass of phenolic compounds mainly contained in herbs, tea, citrus fruits, peas and spinach. The most studied compounds of this subclass include apigenin and luteolin [39]. Apigenin has shown the capacity of inhibiting age-related muscle atrophy in mouse models by reducing oxidative stress and preventing apoptosis of skeletal muscle cells [119]. It can also promote protein synthesis and modulate local inflammation through TNFα downregulation [120,121]. Luteolin, instead, is mainly known for its anti-atherosclerotic properties, inhibiting proliferation and migration of vascular smooth cells in vascular plaques [122]. However, recent evidence suggests that it can suppress inflammation and protein degradation also in skeletal muscle cells, making it a potential therapeutic agent in age-related sarcopenia [123]. Interestingly, the administration of a nutritional supplement consisting in luteolin and the xanthonoid compound mangiferin was associated with improved physical performance and increased oxygen extraction by skeletal muscle cells in a group of young physically trained men [124].

Dietary flavones are subject to gut microbial metabolism. However, the specific pathways are less known than for other phenolic subclasses. The bioavailability of these compounds is largely dependent on the microbial hydrolyzation of glycoside conjugates and C-ring breakdown, leading to the formation of a large number of absorbable compounds exerting physiological functions [98,125]. These steps mainly depend on bacterial functionalities harbored in a limited number of taxa, including *Enterococcus avium*, *Parabacteroides distasonis*, *Eubacterium ramulus* and, most of all, *Flavonifractor plautii* (formerly known as *Clostridium orbiscindens*) [126,127].

The specific role of these gut bacterial species in the gut-muscle axis of older individuals is still unknown. However, *Parabacteroides distasonis* was found as a marker of gut microbiota flexibility in older individuals [128] and was associated with improvements in muscle mass after a sodium–glucose co-transporter-2 inhibitor treatment in obese mice [129]. It is also considered an emerging probiotic for its significant anti-inflammatory properties [130,131]. *Eubacterium ramulus* is known for its capacity of synthetizing butyrate, which is the main SCFA with myoprotective actions, and for its antinflammatory properties [132,133]. *Flavonifractor plautii* abundance has been recently identified as a marker of a healthy diet style, correlating with the dietary intake of legumes, fruit and vegetables [134] and providing protection against arterial stiffiness in aging [135]. It is also characteristically less abundant in the microbiota of older individuals in comparison with adults, according to a study conducted on a sample of 64 healthy subjects from Singapore [136].

Overall, these findings support the hypothesis that older individuals at risk for physical frailty and sarcopenia may have a reduced representation of bacterial functionalities able to perform biotransformation of flavones into active compounds with putative myoprotective action, but no studies have specifically addressed this issue to date. Further studies on the pathways involved in flavone metabolism at the gut microbiota level should be available before this phenolic subclass may be considered as a clinically reliable nutritional supplement against sarcopenia.

### 3.6. Isoflavones

Isoflavones are a class of phenolic compounds with a molecular structure resembling human steroid estrogens and exerting estrogenic or antiestrogenic effects by interaction with the estrogenic receptors Erα and Erβ [40,137]. The most studied isoflavones include daidzein and genistein, mainly contained in soy, and glabridin, which is mainly contained in licorice [40,41].

Daidzein can promote oxidative phosphorylation and fatty acid oxidation in skeletal muscle cells through the activation of ERα, reducing lipid accumulation in muscle tissue [138]. The interaction between daidzein and ERβ can also result in the down-regulation of ubiquitin proteases and inhibition of Glut4/AMPK/FoxO pathway and atrogin-1 expression, resulting in reduced protein degradation and protection against muscle atrophy [139,140]. Finally, daidzein has also a role in promoting mitochondrial biogenesis [141]. On the other side, genistein has demonstrated the capacity of alleviating denervation-induced muscle atrophy through interaction with ERα [142]. Interestingly, in skeletal muscle cells, genistein downregulates the expression of the micro-RNA miR-222, which is characteristically increased in muscle atrophy [143]. This mechanism can lead to muscle regeneration and regulation of muscle fiber type [144,145]. Finally, the isoflavone derived from licorice, glabridin, is able to reduce protein degradation and promote glucose uptake in skeletal muscle cells [146,147].

The administration of soy isoflavones to mouse models of muscle atrophy and mice at risk for cancer-related cachexia resulted in the prevention of muscle wasting [148,149]. Randomized controlled trials testing the effects of soy isoflavone supplementation on body composition of postmenopausal women have provided conflicting results, with one study showing increased muscle mass [150], and another study showing no significant effect on phase-angle bioimpedance analysis [151]. However, the administration of soy isoflavones in combination with whey and soy protein extracts to older individuals resulted in an improvement of inflammation with reduced interleukin-6 levels [152]. Short-term supplementation with soy derivatives was also associated with improved physical performance in endurance athletes [153].

In foods, isoflavones are mainly present in a glycosylated form, which is not absorbable by the intestinal mucosa [27]. Thus, to exert their biological actions, isoflavones must undergo deglycosylation by intestinal brush border β-glucosidase [154]. Bacterial β-glucosidases also contribute to the process in a significant way, increasing the dietary bioavailability of isoflavones [154]. *Lactococcus, Enterococcus*, *Lactobacillus* and, to a lower extent, *Bifidobacterium*, are the main bacterial taxa contributing to this process [155]. Gut microbial communities, with a high representation of these species, should promote genistein and daidzein bioavailability and enhance their biological actions after soy ingestion [156].

Few data are currently available regarding the gut-muscle axis of the most efficient of these bacterial taxa, *Lactococcus* spp., in converting soy isoflavones into absorbable aglycone forms. However, the administration of *Lactococcus cremoris* fermented milk to middle aged mice promoted muscle protein synthesis and contributed to improve muscle mass [157]. Furthermore, the prescription of a high-protein diet targeted against sarcopenia to a group of older women was associated with an increased representation of *Lactococcus* spp. in the gut microbiota [158]. Conversely, as discussed in Section 3.1, *Lactobacillus* is considered as one of the most promising probiotics against sarcopenia with its supplementation being associated with improvements in muscle mass and physical function in both mouse models and human beings [68,159,160]. In particular, the administration of *Lactobacillus paracasei*, leucine and omega-3 fatty acids was particularly effective in improving muscle mass and function in a group of older frail individuals with an average age of 79.7 years old [161]. Observational studies suggest that the microbiota composition of subjects with sarcopenia and physical frailty may be characterized by increased representation of *Lactobacillus* spp. and *Eubacterium* spp. [65,69,82]. This circumstance enables the hypothesis that older sarcopenic individuals may be particularly prone to the myoprotective effects of soy isoflavones, but no specific studies have addressed this issue to date. Therefore, nutritional supplements containing soy isoflavones represent very promising candidates as non-pharmacological treatment against sarcopenia, especially in association with probiotics containing Lactobacilli or Bifidobacteria.

After deglycosylation, isoflavones can either be absorbed into circulation and undergo liver metabolism, or be further transformed by the gut microbiota. Daidzein, in particular, can undergo several biotransformations to equol: a biologically active phytoestrogen with several physiological functions. Several bacterial species are involved in these pathways, including *Lactobacillus*, *Bifidobacterium*, *Clostridium*, *Eggerthella* and *Adlercreutzia*, so that two distinct metabotypes (equol producers and non-producers) can be identified [162]. Conversely, genistein can be transformed into hydroxyphenylpropionic acid through multiple steps involving *Lactococcus*, *Eubacterium ramulus* and, probably, *Butyricimonas* [27,163]. The physiological functions of both equol and hydroxyphenylpropionic acid on skeletal muscle, however, are still unknown. Thus, no hypotheses can be made on the relevance of these biotransformations and the corresponding metabotypes for the pathophysiology of sarcopenia.

### 3.7. Flavonols

The phenolic subclass of flavonols mainly includes rutin, quercetin and morin. The major source of rutin is buckwheat, but it is also present in apples, citrus fruits, asparagus, onions and tea [164]. Experimental mouse studies have shown that rutin is associated with increased protein synthesis and mitochondrial biogenesis, and reduced apoptosis, in skeletal muscle cells [165,166]. Rutin has also shown antinflammatory properties in vitro [167], confirming its potential beneficial effect against muscle wasting.

Quercetin is a rutin derivative that is naturally found in capers, herbs, radish, fennel, onions and berries, or can originate from rutin de-glycosylation carried out by intestinal mucosa enzymes [27,42]. Quercetin is well known for exerting pleiotropic myoprotective actions, being able to stimulate protein synthesis, inhibit apoptosis [168], reduce oxidative stress [169], promote mitochondrial biogenesis [170], regulate fiber type switching [171], promote the myogenic differentiation of stem cells [172], attenuate adipogenesis and fibrosis [173], and regulate motor unit firing patterns [174] in skeletal muscle cells. For these reasons, quercetin supplementation can help to limit muscle damage and promote recovery after strenuous eccentric exercise in adult subjects [175,176,177]. However, the administration of quercetin supplements in combination with resistance low-intensity exercise did not result in improvements in muscle muss, but only in muscle stiffness, in an older group of Japanese community-dwellers [178].

Morin, a less common flavonoid found in osage orange and guava, can also exert myoprotective actions by reducing oxidative stress and inhibiting pro-apoptotic pathways in skeletal muscle cells [179,180,181].

Dietary flavonols undergo relevant biotransformations in the gut lumen through interaction with the gut microbiota [27]. Rutin, in particular, can be transformed into quercetin with substantial contribution of gut microbiome functionalities. According to a recent experimental model, in gut microbial communities, the rate of conversion of rutin into quercetin is positively associated with the abundance of Enterobacteriaceae and Lachnospiraceae, and particularly *Lachnoclostridium* spp. [182]. Interestingly, reduced abundance of *Lachnoclostridium* was recognized as a marker of sarcopenia and physical frailty in the human study by Kang and colleagues [69]. However, other studies reported an increased representation of Enterobacteriaceae in sarcopenic subjects [65,66,82], and the abundance of this family is generally considered a hallmark of age-related dysbiosis, being particularly represented in older frail subjects residing in nursing homes [183,184]. Thus, it is unclear whether the gut microbiota of sarcopenic older individuals exhibits a capacity of biotransforming rutin into quercetin significantly different than that of healthy individuals.

Quercetin, either derived from diet or the biotransformation of rutin, can also undergo further bacterial biotransformations in the gut lumen, resulting in a wide range of compounds, such as homovanillic acid, dihydroxyphenylacetic acid, isorhamnetin and sulfonilated or glucuronated conjugates of quercetin [27]. All these compounds exert biological effects similar to quercetin. The bacteria more frequently involved in such biotransformation pathways include *Eubacterium ramulus*, *Eubacterium oxidoreducens*, *Flavonifractor plautii*, and *Butyrivibrio* spp. [185]. A microbiome rich in these taxa should therefore be associated with enhanced myoprotective effects of quercetin. The putative role of *Eubacterium ramulus* and *Flavonifractor plautii* in the microbiome of older individuals has been discussed in Section 3.5. *Butyrivibrio* depletion, instead, has been recently recognized as a marker of deep dysbiosis in the extreme ages of life, particularly in individuals approaching death [186], and in older patients with Parkinson’s disease [187]. Its abundance was associated with modulation of Th1 and Th2 immune responses and their related inflammation in a group of 688 healthy adults [188]. These findings, albeit very preliminary, suggest that older patients at risk of physical frailty and sarcopenia may have a reduced representation of bacterial functionalities that are able to biotransform quercetin into physiological effectors, but specific studies should assess this hypothesis before recommendations on quercetin supplementation can be made.

Although experimental data suggest that morin has a physiological action similar to quercetin with regard of skeletal muscle, no studies have specifically assessed its interactions with gut microbiota to date.

### 3.8. Anthocyanins

Anthocyanins are a phenolic subclass with a basic flavylium aglycone structure, frequently found in berries, grapes, plums and other vegetals with red or violet pigmentation [43]. Delphinidin and cyanidin are the most studied compounds of this subclass that also includes malvidin, peonidin, petunidin and pelargonidin. These substances exhibit positive physiological effects for humans, especially on arteries and sensory organs [43]. The effects of anthocyanins on skeletal muscle cells have been demonstrated clearly only for delphinidin, which prevents muscle atrophy, promotes protein synthesis, inhibits apoptotic pathways and exerts antioxidant actions [189,190,191]. Anthocyanins extracted from pigmented fruits, however, are well known dietary supplements able to improve the physiological responses to intense exercise, especially by increasing oxygen delivery to myocells, and positively influence the muscular performance in athletes [192,193,194]. Furthermore, a nutritional intervention rich in foods containing cyanidin was also associated with a reduced progression of muscular dystrophy in a recent pilot study [195].

Anthocyanins have a low bioavailability in the human intestinal tract with only small fractions of total dietary intake that can be digested and absorbed in the small intestine [196]. Bioavailability is, instead, consistently increased by interaction with the gut microbiota [196]. In the colon, anthocyanins undergo hydrolysis of their sugar moieties by bacterial enzymes. The aglycone forms are then transformed into a wide variety of compounds, including protocatechuic acid, vanillic acid and gallic acid [197]. Cyanidin, in particular, is consistently transformed into protocatechuic acid, which, according to a recent experimental study, exhibits several myoprotective actions, including the reduction of oxidative stress, promotion of mitochondrial biogenesis and conversion of skeletal muscle fibers from type II to type I [198]. The delphinidin derivative, gallic acid, has instead shown anti-sarcopenic properties in in vitro studies, where muscle tissues were incubated with vegetal extracts [199,200,201].

The precise bacterial taxa involved in transformation of anthocyanins into protocatechuic or gallic acid are still unknown. The enzymatic functionalities needed for these pathways may be harbored in several taxa of the genera *Bacteroides*, *Clostridium* and *Eubacterium* [196,202]. Other in vitro studies suggest that different microbiota composition may be associated with different pathways of biotransformation of anthocyanins, in some cases with beneficial physiological activities, and in other cases with unknown effects for the host [203,204]. For example, the incubation of an anthocyanin-rich elderberry extract with three different bacteria commonly found in the human microbiota (*Enterobacter cancerogenous*, *Bifidobacterium dentium* and *Dorea longicatena*) was associated with extreme variety of final metabolic products [204], suggesting that the anthocyanin–microbiota interaction could be extremely variable across individuals and not classifiable in a limited number of metabotypes. However, extreme levels of dysbiosis, which are frequently found in older individuals with sarcopenia [14], may be associated with an impaired capacity of producing gallic and protocatechuic acids, the main effectors of beneficial actions of anthocyanins on skeletal muscle. Therefore, the putative anti-sarcopenic effects of dietary anthocyanins could suffer from an extreme inter-individual variability of physiological responses depending on gut microbiota composition and functionality. Since aging is characterized by a significant increase in the inter-individual variability of gut microbiota composition and functionality, anthocyanin supplementation does not represent, at the current state of knowledge, a good candidate for developing novel nutraceuticals against sarcopenia, because the responses to treatment have a high risk of being extremely variable and unpredictable.

### 3.9. Resveratrol

Resveratrol is the most common and known compound belonging to the phenolic subclass of stilbenes [27]. It is synthetized by plants as an answer to stressful conditions, and this circumstance makes its concentration in foods extremely variable [27]. Grapes, berries and peanuts are the foods with the average higher content of resveratrol, but it can be found also in other fruits or vegetables, such as banana, pineapple, peach, apple, pear, potato and cucumber [44]. The *trans-* isomer of resveratrol is responsible for most of its biological actions, which has been extensively studied in vitro and in experimental models [205]. Basically, it exerts powerful anti-oxidant, anti-inflammatory and cytoprotective actions through the activation of SIRT1, and the promotion of mitochondrial functions in target cells [206,207].

The activation of sirtuins (SIRT1) and their related signaling pathways are deemed to be of pivotal importance for the prevention of age-related sarcopenia, because they promote mitochondrial biogenesis and function, and ultimately, favor protein synthesis and delay apoptosis in skeletal muscle cells [208]. Sirtuins are considered an emerging therapeutical target in sarcopenia, and the circumstance that resveratrol is a strong activator of SIRT1 has boosted research on the putative anti-sarcopenic action of resveratrol [207]. The incubation of murine myoblasts with resveratrol was in fact associated with a resistance to apoptosis even after the exposure to oxidative stress [209]. In murine models of sarcopenia and sarcopenic obesity, the administration of resveratrol was associated with improvements in muscle mass and function, and, at the cellular level, with increased mitochondrial biogenesis and reduced apoptosis [210,211,212]. These effects may be particularly emphasized for glycolytic white muscular fibers, and only of moderate extent for red fibers [213]. Resveratrol treatment was also associated with reduced markers of skeletal muscle inflammation in mice [214]. Interestingly, a randomized controlled trial conducted in middle-aged men with metabolic syndrome showed that resveratrol treatment was also associated with the increased levels of markers of muscle turnover [215]. These effects may be synergistically enhanced when resveratrol supplementation is associated with exercise treatment programs, causing significant improvements in muscle strength in both animal models and human beings [212,216,217].

Despite this evidence, other reports put into question the beneficial anti-sarcopenic effects of resveratrol in both mouse models and human beings. Three studies failed to detect significant improvements in muscle mass and function after the administration of this compound to aged rats, even if the oxidative stress burden was reduced [218,219,220]. Resveratrol was also unable to induce a significant hypertrophic response with the activation of muscle satellite cells in older mice [221]. In human beings, the administration of resveratrol as a nutritional supplement was associated with only minor improvements in muscle mass and function [222], and with negligible effects on chronic low-grade inflammation [223].

As for other phenolic compounds, the bioavailability and biological activity of resveratrol are deeply influenced by the gut microbiota [27]. Dietary resveratrol can be absorbed in the small intestine without undergoing biotransformation, and is then subject to hepatic glucuronidation or sulfation to form active metabolites [224]. A significant portion of dietary resveratrol, however, reaches the colon and is subject to bacterial metabolism. Two major pathways have been identified. First, resveratrol can be hydroxylated to the bioactive form dihydroresveratrol by bacterial taxa harboring specific enzymatic functionalities, including *Adlercreutzia equolifaciens* and *Slackia equolifaciens* [224,225]. As discussed in Section 3.3, *Adlercreutzia equolifaciens* is particularly abundant in the fecal microbiota of subjects with a sedentary lifestyle [103], suggesting that individuals with these characteristics could be particularly prone to the beneficial effects of resveratrol. *Slackia equolifaciens*, instead, was associated with the body fat content in a group of patients with cirrhosis [102] and was significantly enriched in the fecal microbiota composition of patients without sarcopenia suffering from heart failure [226].

Another microbial metabolic pathway involves the transformation of resveratrol into 3,4′-dihydroxy-trans-stilbene and 3,4′-dihydroxybibenzyl (lunularin) [227]. According to a recent study conducted in mice, these microbiome-derived metabolites of resveratrol account for a significant part of its biological effects, exhibiting even stronger anti-inflammatory effects than its progenitor [228]. Interestingly, Iglesias–Aguirre and colleagues have recently shown that two distinct microbiome metabotypes, with regard to lunularin production, exist in human beings with just 74% of a group of 195 healthy volunteers able to produce lunularin after resveratrol ingestion, due to specific gut microbiome functionalities [229]. In another study, Jarosova and colleagues identified elevated inter-individual variability in the resveratrol microbial metabolism after the incubation of resveratrol extracts with fecal cultures of different human donors [230]. The taxa harboring these functionalities, however, were not identified in any of these studies.

Overall, the current, state-of-the-art literature suggests that the resveratrol-microbiome interaction may be much more complex that what initially supposed, and that the microbiome may contribute to explain a substantial part of the inter-individual variability of responses after resveratrol administration, especially in the context of older individuals. Therefore, despite resveratrol being among the most studied phenolic compounds, the state of knowledge on its biotransformation at the gut microbiome level in older individuals suggests caution in considering it as a promising treatment against sarcopenia.

### 3.10. Lignans

Lignans are a subclass of polyphenols with a steroid-analogous chemical structure, found in herbs typically used in a Chinese traditional diet [45,46]. Schisandrin A, magnolol and sesamin are the most known compounds of this class, which includes a large number of molecules [45,46]. Lignans exert anti-inflammatory and antioxidant actions, and can also mimic estrogenic effects due to their particular chemical structure [231]. Both schisandrin A [232] and magnolol [233] prevent muscle wasting in mouse models of drug-induced sarcopenia. The highest myoprotective actions, however, have been observed for sesamin, which is able to extend lifespan in *Caernohabditis elegans* [234], reduce aging phenotypes in *Drosophila* muscles [235], maintain exercise capacity and mitochondrial function in mice fed a high-fat diet [236], and promote myocellular vitality by activating the sirtuin pathway and inhibiting irisin synthesis [237]. To date, no study has tested the effects of these compounds on the skeletal muscle mass and function of human beings.

As for other polyphenol subclasses, the biological effects of dietary lignans are consistently mediated by the gut microbiota [238]. Enterodiol and enterolactone, the so-called enterolignans, have been identified as the major products of complex biotransformative pathways when lignans are incubated with the fecal microbiota from human donors [239]. The synthesis of these compounds, however, suffers from a significant inter-individual variability in vivo [240], so that three different metabotypes could be identified (low, middle and high producers) [241]. Age is significantly associated with the low producer phenotype, according to the results of three different studies [242,243,244], probably as a result of gut microbiota alterations associated with ageing.

Multiple microbial species may be involved in the synthesis of enterolignans because different functionalities may act at different metabolic steps. *Bacteroides* and *Clostridium* spp. can promote lignan de-glycosylation [245]; these taxa are frequently well-represented in the gut microbiota, so that this does not appear to be the limiting step of the biotransformation pathway. *Eubacterium limosum*, *Blautia producta*, *Eggerthella lenta* and *Acetobacterium dehalogenans* could instead be the key taxa involved in the further steps [246]. Interestingly, as discussed in Section 3.3, *Eggerthella lenta* has been recognized as a marker of frailty in several studies, being positively associated with sarcopenia [35,101,102,103]. *Eubacterium* spp. is another marker of physical frailty and sarcopenia [65,69,82], while the abundance of *Blautia* spp. was positively associated with appendicular lean mass and the presence of malnutrition [67,247,248]. Therefore, the capacity of transforming lignans to bioactive enterolignans in older individuals may particularly rely on the interaction between these bacterial taxa and their relative abundance.

### 3.11. Curcumin

Curcumin is a phenolic compound derived from the rhizome of turmeric and ginger, exhibiting pleiotropic physiological effects, including antioxidant, anti-inflammatory, anti-cancer, antimicrobial and hypoglycemic actions [47]. The antioxidant and anti-inflammatory properties of curcumin have been exploited in skeletal muscle medicine as a means of facilitating recovery after strenuous physical exercise [249], and for improving performance in physically active individuals [250,251,252]. In a randomized controlled trial, curcumin administration was associated with reduced circulating cytokine and creatine kinase levels in healthy adults with a lower perception of muscle soreness [253]. These effects depend on a direct effect of curcumin on skeletal muscle cells, improving post-exercise lactate accumulation [254], reducing protein breakdown [255], and stimulating protein synthesis through the sirtuin-3 pathway [256]. Furthermore, in older subjects, curcumin may promote the optimal microvascular perfusion of skeletal muscles [257]. Multiple studies conducted in animal models of sarcopenia have shown that oral administration of curcumin is associated with the reduction of muscle wasting and improvements in markers of inflammation and oxidative stress [258,259]. Similar effects were also obtained with parenteral administration [260]. The beneficial effects, however, were less pronounced for long-term supplementation because curcumin also caused reduced food intake [261]. In a randomized controlled trial conducted on thirty older healthy subjects, the administration of a curcumin supplement was associated with significant increases in muscle performance of both lower and upper limbs [262].

Curcumin is scarcely bioavailable after an oral load [27]. The absorbed fraction undergoes extensive phase I and phase II metabolism in the intestine and liver, forming hydroxylated or glucuronated metabolites that are responsible for the biological actions of curcumin [27]. Recent studies, however, have contributed to elucidate that, like many other phenolic compounds, curcumin is extensively metabolized by the gut microbiota, forming hydroxylated and methylated derivatives that exert the same biological functions of their progenitor [263,264,265]. *Escherichia coli* and *Blautia* spp. seem to be the key bacterial taxa involved in these metabolic pathways, contributing to significantly enhancing the bioavailability of curcumin after an oral administration [263,264]. However, no specific metabotypes of curcumin metabolization by the microbiota have been investigated to date, and the relevance of microbiota composition for curcumin bioavailability remains speculative in clinical terms.

It is noteworthy, however, that both of the bacterial taxa involved have a known association with skeletal muscle mass in older individuals, according to the current, state-of-the-art literature. As discussed in Section 3.10, the fecal microbiota abundance of *Blautia* was associated with appendicular lean mass in two studies [67,247]. The abundance of *Escherichia coli* was positively associated with the lumbar skeletal muscle index in a group of patients suffering from cirrhosis [266]. These circumstances suggest that the myoprotective effects of curcumin may be particularly emphasized in those subjects with sufficient microbiota representation of these two taxa.

## 4. Discussion and Perspectives

All the phenolic subclasses listed in Table 1 and extensively discussed in Section 3 exhibit protective effects on skeletal muscle cells and tissues, which may potentially be effective as part of a therapeutical strategy for counteracting sarcopenia in the older age. However, the effects of these compounds in vivo are much less clear, and rigorous randomized controlled trials testing the effect of supplementation with one or more phenolic compounds on clinical endpoints related to skeletal muscle mass and function are available only for a very small number of substances. Furthermore, virtually no human study was conducted in an oldest old population, the one with the highest risk of physical frailty and sarcopenia. Such circumstance suggests caution in transferring data collected in vitro or in mouse models to clinical practice.

**Figure 1:**
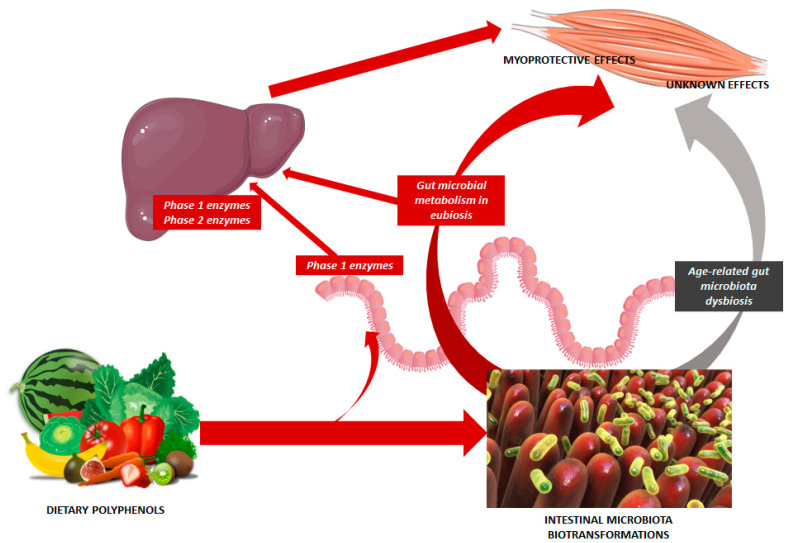
Model of interaction between dietary polyphenols and intestinal gut microbiota, and its consequences for the bioactive effects counteracting muscle wasting. The healthy gut microbiota can improve the bioavailability of phenolic compounds, and contribute to enhancing the protective actions for skeletal muscles. Conversely, the effects of the interaction between dietary polyphenols and dysbiotic microbiota in older individuals are still unknown.

The interaction between polyphenols and intestinal microbiota could consistently increase the variability of physiological responses to dietary polyphenol supplementation, especially in older individuals who are particularly prone to gut microbiota dysbiosis [50,51,267]. As summarized in Figure 1, all phenolic subclasses seem to exert several of their myoprotective actions even without the contribution of gut microbiota [27]. In fact, a certain degree of intestinal absorption and biotranformation by intestinal and liver phase I and II enzymes can be detected for all compounds [27]. However, the individual microbiota composition, and the related metabolic functionalities of bacteria, may consistently modify the bioavailability and bioactivity of dietary phenolic compounds, leading to extremely variable physiological responses across individuals (Figure 1) [268,269]. The presence of specific bacterial functionalities may in fact favor the synthesis of bioavailable and bioactive mediators in an individual manner [268,269]. Distinct “metabotypes” can be identified for some phenolic subclasses, such as ellagitannins and curcumin, but, in most cases, the interaction between phenolic compounds and gut microbiota is so complex that no standardized responses could be identified in the existing studies. In any case, the comprehension of this interaction has consistently improved in the last decade [270,271], and the biochemical pathways are being increasingly understood [272]. The key taxa involved in the biotransformation of each phenolic subclass, according to the current state of knowledge, are listed in Table 2.

In this scenario, studies assessing the effect of polyphenol supplementation as a possible treatment strategy against sarcopenia should mandatorily account for the mediatory effect of the gut microbiota, as recently recognized in the field of dementia [273]. Furthermore, a deep knowledge of biochemical pathways involved in gut microbial biotransformation of phenolic compounds could lead to the development of personalized nutritional intervention approaches against physical frailty and sarcopenia. In fact, the hallmark of the aging gut microbiota is the increase of inter-individual variability within each population, with enhanced differences in the overall architecture of microbial communities, even in the presence of a similar phenotype and pattern of aging [12,13,28]. This is particularly true for the oldest old patients with a high burden of multimorbidity and polypharmacy, who generally exhibit disruption of gut microbial communities with an overrepresentation of opportunistic pathogens [274,275]. Thus, a polyphenol-based nutritional intervention that exhibits beneficial effects against physical frailty and sarcopenia in one individual may not be necessarily effective in another individual, due to different interactions with the microbiota. Furthermore, the use of combined interventions, comprising the administration of probiotics and phenolic supplements, should be investigated in the future, and the knowledge of the interaction between the microbiota and each phenolic subclass should be paramount for designing such interventions [276].

Dietary interventions increasing the intake of polyphenols and the administration of polyphenol-based nutritional supplements also have favorable effects on the gut microbiota structure [268,269]. A beneficial modulation of the gut microbiota has been described for almost all phenolic subclasses. Although a comprehensive discussion of this aspect surpasses the purposes of the present review, it should be carefully considered in all studies assessing the beneficial muscular effects of dietary polyphenols as well. However, no studies have specifically assessed the clinical effects of polyphenol supplementation in older human beings to date.

We acknowledge that this narrative review has several limitations because it is mainly based on the analysis and discussion of evidence not obtained from studies conducted in patients with sarcopenia, but from in vitro investigations, animal studies or from populations of healthy human beings. Thus, the concepts discussed mainly represent hypotheses that need further investigation and validation. Furthermore, the role of gender-specific differences in gut microbiota and phenolic compound metabolism has not been considered. Phenolic compounds could also have different physiological effects on skeletal muscle cells at different concentrations. Thus, when investigating each (poly)phenol subclass as a potential nutraceutical treatment strategy against sarcopenia, pharmacokinetic issues should be considered. Unfortunately, the state of knowledge on this topic did not allow us to include pharmacokinetic issues in our discussion. Finally, the framework of this review was tailored to age-related or chronic disease-related sarcopenia, and did not consider alternative causes of muscle wasting, such as prolonged immobilization, severe stroke, and primary myopathies such as muscle dystrophy.

Despite these limitations, the current state of knowledge indicates clear lines of future research for improving the understanding of the complex interaction between dietary intake of polyphenols, gut microbiota and muscle health in aging individuals.

## 5. Conclusions

Phenolic compounds with putative beneficial actions against age-related sarcopenia exhibit a substantial interaction with gut microbial communities that modifies their bioavailability and bioactivity. This effect may be particularly emphasized in older individuals who frequently exhibit an age-related dysbiosis of gut microbiota and increased inter-individual variability of bacterial community structure and functions. The interaction between polyphenols and the gut microbiota should be carefully considered in the design of studies and therapeutical interventions aimed at counteracting the burden of sarcopenia.

## Figures and Tables

**Table 1:** Overview of the main phenolic compounds that exhibit potential myoprotective action in vitro or in experimental models of sarcopenia/muscle damage. The main dietary sources of relevance for human nutrition are also indicated for each compound. Information provided in Table are summarized from the review article by Nikawa and colleagues [24] and other manuscripts focused on dietary sources of phenolic compounds and mechanisms of interaction with the host [30,31,32,33,34,35,36,37,38,39,40,41,42,43,44,45,46,47].

Polyphenol Class	Polyphenol Subclass	Compound	Main Dietary Sources	Action in Experimental Models
Phenolic Acid	Hydroxybenzoic Acid	Gallic Acid	Berries, plums, grapes, mango, tea, wine	Increased mitochondrial function and biogenesis
		Ellagic Acid	Berries, grapes, pomegranates, walnuts	Induction of antioxidant enzymes, protection against mitochondrial dysfunction
		Urolithin A	Berries, grapes, pom-egranates, walnuts	Increased muscle angiogenesis, energetic capacity and contractile function
		Urolithin B	Berries, grapes, pom-egranates, walnuts	Increased protein synthesis, myotube differentiation and muscular fiber hypertrophy
	Hydroxycinnamic Acid	Ferulic Acid	Rice, wheat, oats, beans, coffee, artichoke, nuts	Regulation of muscle fiber differentiation and stimulation of myogenic transcriptional factors
		Chlorogenic Acid	Apples, artichoke, coffee, grapes, pears, kiwi, plums, potatoes	Improvement of mitochondrial function and energy metabolism
		Caffeic Acid	Coffee, olives, carrots, potatoes, fruits	Stimulation of myocellular differentiation and hypertrophy
Flavonoids	Flavanols	Epicatechin	Berries, grapes, wine, cocoa, plums, tea	Induction of mitochondrial biogenesis and myogenic differentiation; decreased follistatin and myostatin
		Epigallocatechin	Berries, grapes, wine, cocoa, plums, tea	Upregulation of myogenic transcriptional factors, antioxidant
		Epigallocatechin Gallate	Berries, grapes, wine, cocoa, plums, tea	Reduction of protein degradation, reduction of proapoptotic signaling, inhibition of NF-κB
	Flavanones	Hesperidin	Citrus fruits	Increased mitochondrial function, reduced oxidative stress
		Naringenin	Citrus fruits	Increased glucose uptake, regulation of skeletal muscle cell differentiation
	Flavones	Apigenin	Herbs, tea, wine, citrus fruits, spinach, broccoli, peas	Inhibition of mitophagy and autophagy, enhanced myogenic differentiation, downregulation of TNFα
		Luteolin	Herbs, tea, wine, citrus fruits, spinach, broccoli, peas	Downregulation of pro-inflammatory cytokines, antioxidant
	Isoflavones	Genistein	Soybeans, fava beans, lupin, kudzu, psoralea, coffee	Inhibition of apoptosis, increased myocellular differentiation, antioxidant
		Daidzein	Soybeans, tofu, kudzu	Inhibition of protein degradation, promotion of myocellular differentiation
		Glabridin	Licorice	Inhibition of protein degradation
	Flavonols	Quercetin	Capers, herbs, coriander, radish, fennel, onion, radicchio, berries	Reduction of myostatin, antioxidation, increased mitochondrial biogenesis, reduction of protein degradation
		Morin	Osage orange, guava	Antioxidation, reduction of protein degradation
	Anthocyanins	Delphinidin	Berries, pomegranates, grapes	Antioxidation, reduced atrogin-1 expression and protein degradation
		Cyanidin	Grapes, berries, cherry, apple, plum	Reduced inflammation and fibrosis
Polyphenol	Stilbene	Resveratrol	Grapes, berries, peanuts	Reduction of atrogin-1, reduction of oxidative stress, improvement of mitochondrial function, inc5reased protein synthesis, regulation of mTOR signaling, induction of myotube hypertrophy
	Lignan	Schisandrin A	Shengmainsan (Chinese traditional herb)	Suppression of protein degradation and stimulation of protein synthesis
		Magnolol	Magnolia bark	Stimulation of IGF-1 mediated protein synthesis
		Sesamin	Sesame	Reduced oxidative stress, increased mitochondrial function
Other	Curcumin	Curcumin	Turmeric, ginger, food additives	Inhibition of atrogin-1, reduction of oxidative stress, promotion of myofibrillar differentiation, reduction of proteasome expression and protein degradation

**Table 2:** Overview of bacterial taxa involved in microbial metabolism for each phenolic subclass.

Phenolic Subclass	Bacterial Taxa Involved in Gut Microbiota Biotransformation Pathways	Metabotypes Identified
Ellagitannins	*Akkermansia muciniphila*	Yes (UroA, UroB, Uro0)
	*Gordonibacter* spp.	
	*Eggerthellaceae*	
	*Lactobacillus* spp.	
	*Leuconostoc* spp.	
	*Pediococcus* spp.	
Chlorogenic acid and derivatives	*Bifidobacterium* spp.	No
Flavanols/Proanthocyanidins	*Clostridium coccoides*	No
	*Bifidobacterium infantis*	
	*Eggerthella lenta*	
	*Adlercreutzia equolifaciens*	
Flavanones	*Bifidobacterium* spp.	Yes (hesperidin producers or not)
	*Eubacterium limosum*	
	*Eubacterium ramulus*	
Flavones	*Enterococcus avium*	No
	*Parabacteroides distasonis*	
	*Eubacterium ramulus*	
	*Flavonifractor plautii*	
Isoflavones	*Lactococcus* spp.	Yes (equol producers/non producers)
	*Enterococcus* spp.	
	*Bifidobacterium* spp.	
	*Clostridium* spp.	
	*Eggerthella* spp.	
	*Adlercreutzia* spp.	
	*Butyricimonas* spp.	
	*Eubacterium ramulus*	
Flavonols	*Lachnoclostridium* spp.	No
	*Eubacterium ramulus*	
	*Eubacterium oxidoreducens*	
	*Flavonifractor plautii*	
	*Butyrivibrio* spp.	
Anthocyanins	*Bacteroides* spp.	No
	*Clostridium* spp.	
	*Eubacterium* spp.	
Resveratrol	*Adlercreutzia equilifaciens*	Yes (lunularin producers/non producers)
	*Slackia equolifaciens*	
Lignans	*Bacteroides* spp.	Yes (low, middle or high metabolizers)
	*Clostridium* spp.	
	*Eubacterium limosum*	
	*Blautia producta*	
	*Eggerthella lenta*	
Curcumin	*Escherichia coli*	No
	*Blautia* spp.	

## Data Availability

No original data are associated with this manuscript.
